# Starvation and stress: no place to call home

**DOI:** 10.1093/conphys/coz047

**Published:** 2019-08-26

**Authors:** Sean Tomlinson

**Affiliations:** 1 School of Molecular and Life Sciences, Curtin University, GPO Box U1987, Perth, WA, Australia; 2 Kings Park Science, Department of Biodiversity, Conservation and Attractions, Kattij Place, West Perth, WA, Australia

‘Stressed’ means different things to different people. To some, it means ‘desserts’ spelled backwards. To others, it represents a pathological situation caused by external pressures. To ecological physiologists, however, stress is a key component of the physiological feedback loops that allow animals to interact with their environments. Tecot *et al.* (2019) found that lemurs (*Propithecus diadema*) generally had high stress levels during seasons with low food availability, which would be expected. Alarmingly, however, lemurs living in habitats fragmented by logging activities no longer exhibited stress when food was scarce.

Actually, all animals are constantly under some kind of stress. Stress is the signal that causes physiological changes in animals in response to their environment. Under chronic stress, however, stress signals can become washed out to the point where the animal either does not respond or responds inappropriately. This can lead to reproductive failure, for example, and ultimately contribute to population declines. Measuring these stress signals can indicate how challenging an environment is to an animal and can also indicate how environmental change can make things harder, suggesting a ‘breaking point’ when the animal cannot cope.

Tecot *et al.* measured the levels of stress hormones in the feces of lemurs to understand how fragmentation resulting from logging might be adding to their natural stress levels. Lemurs were most stressed during seasons of very low food availability and quality, which makes sense because starvation is stressful. Counter-intuitively, seasonal patterns of stress caused by food scarcity were most obvious in lemurs from undisturbed forests. Essentially, starvation was more stressful to lemurs from undisturbed habitats than to lemurs from logged forests.

So, does forest fragmentation stress lemurs to the point that their health declines?

While Tecot *et al.* found that forest fragmentation caused changes in lemurs and their environments that are often related to stress, such as reduced food availability and impaired body condition, they did not find high levels of stress hormones in these lemurs. In the face of these counter-intuitive results, Tecot *et al.* remind us that all the usual bets are off in populations that are chronically stressed. Activating stress pathways and producing these hormones can be energetically expensive. Chronically stressed animals do not have extra energy to spare. Lemurs from fragmented forests appear to be starved to the extent that they can no longer mount a stress response. As a result, these lemurs are less able to respond to further changes in their environments.

Stress is a difficult thing to manage from a conservation standpoint because it can be hard to determine what is going to push a species to its breaking point. We have all been in situations where things just accumulate, and then something seemingly insignificant pushes us over the edge. Sometimes this results in a little bit of a tantrum, but sometimes it results in something much more serious. Natural systems intrinsically cause fluctuating stress levels as food availability changes, but starvation caused by human activities, such as deforestation and fragmentation, pushes animals closer to their breaking point. Understanding where the populations are on this continuum is a critical new frontier in conservation physiology.

Illustration by Erin Walsh; Email: ewalsh.sci@gmail.com

**Figure:**
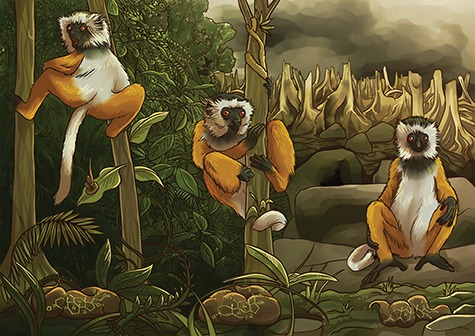


## References

[ref1] Tecot SR, Irwin MT, Raharison J-L (2019) Faecal glucocorticoid metabolite profiles in diademed sifakas increase during seasonal fruit scarcity with interactive effects of age/sex class and habitat degradation. *Conserv Physiol* 7: coz001; doi:10.1093/conphys/coz001. 30746150 PMC6364291

